# Community-Initiated Breast Cancer and Environment Studies and the Precautionary Principle

**DOI:** 10.1289/ehp.7784

**Published:** 2005-03-31

**Authors:** Julia Green Brody, Joel Tickner, Ruthann A. Rudel

**Affiliations:** 1 Silent Spring Institute, Newton, Massachusetts, USA; 2 Department of Community Health and Sustainability, University of Massachusetts, Lowell, Massachusetts, USA

**Keywords:** breast cancer, community-based participatory research, endocrine-disrupting compounds, environmental exposure assessment, geographic information system, precautionary principle, public involvement

## Abstract

The precautionary principle implies the need for research paradigms that contribute to “strength of the evidence” assessments of the plausibility of health effects when scientific uncertainty is likely to persist and prevention is the underlying goal. Previous discussions of science that inform precautionary decision making are augmented by examining three activist-initiated breast cancer and environment studies—the Long Island, New York, and Cape Cod, Massachusetts, studies and the National Institute of Environmental Health Sciences breast cancer and environment centers. These studies show how the choice of research questions affects the potential of results to inform action. They illustrate a spectrum of public involvement, population- and individual-level epidemiologic study designs, and the crucial importance of developing and applying new exposure assessment methods. The exposure studies are key because they are critical in assessing plausibility (without exposure to a causal agent, there is no health effect), are prerequisite to health studies, and identify preventable exposures that could be reduced by precautionary policies, even in the absence of strong evidence of harm. The breast cancer studies have contributed to environmental and biological sampling programs for endocrine-disrupting compounds in drinking water and household air and dust and the application of geographic information systems for surveillance and historical exposure assessment. They leave unanswered questions about when to invest in large epidemiologic studies, when negative results are sufficient, and how to pursue ambiguous positive results in further research and policy.

More than 200,000 new cases of invasive breast cancer and 55,000 cases of *in situ* disease are diagnosed annually in the United States (American Cancer Society 2003), and U.S. women’s lifetime risk of breast cancer has doubled from about 1 in 14 in the 1960s to 1 in 7 today, or 1 in 6 including *in situ* disease (Ries et al. 2004). Incidence continues to rise incrementally in the United States (Ries et al. 2004), and it is increasing more rapidly in developing nations (Parkin et al. 2001). High incidence makes breast cancer an urgent public health priority, and because an increased risk over just one generation must reflect modifiable change rather than inherited genes, incidence patterns also signal that breast cancer is a realistic target for prevention. Further evidence comes from a study of women with high-risk *BRCA1* and *BRCA2* genetic variations that showed that 24% of women born before 1940 were diagnosed with breast cancer by age 50, compared with 67% of women born later, indicating that modifiable factors affect even women at high genetic risk (King et al. 2003).

Factors affecting estrogen and progesterone are among the best-established risk factors for the disease. These include age at menarche and menopause, parity, age at first full-term pregnancy, weight gain after menopause, hormone replacement therapy, lack of physical activity, and alcohol use (Bernstein 2002). These effects, although relatively weak, consistently appear in many epidemiologic studies, leading to high confidence in their roles as risk factors. A much more limited inquiry into chemical exposures as breast cancer risk factors provides a new hypothesis for study: Laboratory animal and cell studies support the hypothesis that animal mammary carcinogens and chemicals that mimic estrogen or otherwise disrupt hormones may increase breast cancer risk, just as endogenous and pharmaceutical hormones do (Brody and Rudel 2003; Davis et al. 1993; Wolff et al. 1996). Exposures to mammary carcinogens and endocrine-disrupting compounds (EDCs) are common from sources such as gasoline, pesticides, detergents, plastics, home furnishings, personal care products, and air and water pollution (Brody and Rudel 2003; Rudel et al. 2003).

In the early 1990s, a number of breast cancer activist organizations began pursuing research into these environmental pollutants as possible avenues to breast cancer prevention (Brown et al., in press; McCormick et al. 2003). They won Congressional legislation mandating the Long Island Breast Cancer Study Project (LIBCSP) (U.S. Congress 1993), founded Silent Spring Institute as an independent organization dedicated to breast cancer and environment research (Brody et al. 1996), and later initiated a multicenter National Institute of Environmental Health Sciences (NIEHS) program of research into environmental factors in the course of puberty in girls (NIEHS 2003).

The design of epidemiologic studies to address activist concerns is problematic, however, particularly because the exposure assessments themselves pose challenges (Brody and Rudel 2003). Self-reports, the basis for exposure classification in studies of most known breast cancer risk factors, are at best a weak method for assessing exposure to many pollutants. Randomized clinical trials, the source of most knowledge about the effects of exogenous chemicals on breast cancer, effectively measure pharmaceutical exposures but are not an ethical option for exposures from pollution, work-places, or consumer products. Biomarkers of exposure and sampling methods for environmental media, such as air, water, and food, have been developed for relatively few of the many chemicals hypothesized to affect breast cancer. They are expensive to use in studies large enough to detect risks of the magnitude (probably < 2-fold) we would expect for EDCs based on the relative risks for known hormonal risk factors. Also, they are difficult to apply across the life span, a problem because higher breast cancer risk is associated with hormonal exposures beginning *in utero* (e.g., twinning and maternal diethylstilbestrol use) and extending to within 5 years of diagnosis (e.g., pregnancy and hormone replacement therapy). Finally, strategies for aggregating effects of mixtures have yet to be developed.

Given these challenges, as activist-generated breast cancer research unfolded, tensions emerged from the mismatch between what investigators can achieve through prevailing epidemiologic research paradigms and what activists had hoped to accomplish in time to help their daughters. Lessons from these conflicting perspectives carry many parallels with, and can inform, other health issues for which relevant exposures are similarly difficult to assess and where disease has multifactorial causation (e.g., asthma and learning disabilities). In this commentary, we seek to draw out these lessons by discussing the relationship between activist goals and scientific methods in reference to the precautionary principle, because it provides a framework for generalizing from the breast cancer studies to other public health issues where scientific uncertainty is likely to persist. We focus particularly on the Cape Cod Breast Cancer and Environment Study, because two of us have been involved for nearly a decade in its development and implementation (J.G.B. as principal investigator and R.A.R. as co-investigator for toxicology and environmental science). We also comment on the Long Island study and the NIEHS breast cancer and environment research centers program. The Bay Area Breast Cancer and the Environment Study Group and research in Marin County, California, which offers another early example, is now affiliated with the NIEHS centers program.

## Precaution as a Guide to Environmental Health Research Design

The precautionary principle calls for preventive action in the face of uncertain but suggestive evidence of risk, especially when safer alternatives exist. The 1998 Wingspread Statement on the Precautionary Principle (Raffensperger and Tickner 1999) identifies four central components of precautionary policies: *a*) taking preventive action in the face of uncertainty, *b*) placing responsibility on those who create risks to study and prevent them, *c*) considering alternatives to potentially harmful activities, and *d*) increasing public participation and transparency in decision making. In contrast, current U.S. chemical regulations require substantial evidence of harm before regulatory action is taken, regardless of the availability of alternatives.

Previous discussions have outlined how the precautionary principle calls for changes in research process, questions, interpretations, and policy applications (Kriebel et al. 2001; Stirling and Gee 2002; Tickner 2002, 2003). By approaching public health policy with a greater willingness to act in the face of uncertainty, the precautionary principle expands the scope of relevant science and increases the utility of evidence about hypothesized harms even when that evidence is far from definitive. It calls for assessment of the “strength of evidence” that accrues from a broadly defined toolbox of methods that includes typical hypothesis-testing epidemiologic designs and extends to hypothesis-generating epidemiology, toxicology, exposure assessment, risk assessment, wildlife studies, and human case reports. Precautionary science seeks integrative methods to deal with chemical mixtures and multiple health effects from the same exposure. It implies an iterative process of research and policy making with an explicit role for judgment, which in turn argues for democratization.

Many breast cancer activist organizations, including groups involved in the Long Island and Cape Cod studies, have explicitly endorsed the precautionary principle {examples come from New York (Huntington Breast Cancer Action Coalition 2003), California (Breast Cancer Action 2004), Massachusetts [Massachusetts Breast Cancer Coalition (MBCC) 2004], and Oregon (Crumpacker 2002)}. The history of community-initiated breast cancer studies reflects the influence of the activists’ precautionary thinking on expanding the scope of research and strategies for public involvement.

## Public Participation in Decision Making

Increased public participation and transparency in decision making are a logical starting point for applying the precautionary principle to breast cancer studies because democratizing scientific research opens the door for activists’ priorities to influence study design. Breast cancer activists, following the example of AIDS activists, have become leaders in helping to drive research agendas by catalyzing federal and state legislation and appropriations and participating in research design (Brown et al. 2000; McCormick et al. 2003, 2004). In 1993 and 1994, Long Island and Massachusetts activists initiated unprecedented public roles in research by seeking empowerment in study design and implementation. Frustrated that decades of the war on cancer had not addressed their questions about environmental factors and prevention, both groups circumvented traditional federal grant making and sought help through elected officials. Long Island activists generated the first large-scale breast cancer and environment research through a congressional mandate, and the Cape Cod study, funded by the state legislature, pioneered activist governance in research.

### Long Island study.

The LIBCSP was the first of the activist-generated studies to become nationally visible. Mandated by Congress in 1993, it grew to encompass > 10 studies totaling > $30 million. Beyond winning funds, the Long Island activists specified in the Congressional mandate several aspects of the research design, including a case–control study using biologic markers and the development of a geographic information system (GIS) (U.S. Congress 1993). The grants were then awarded to academic scientists, however, and activists sometimes felt shut out of the process (McCormick et al. 2003). For example, conflict emerged about the list of environmental pollutants under study, with activists advocating for a more extensive set of target compounds (Balaban B, personal communication). The academics, motivated partly by the limited availability of biomarkers for historical exposure, chose to study organochlorines that had been banned in the United States, generating data that would not directly inform current environmental health policy. In addition, hopes that the GIS would allow activists to extend community-based mapping efforts were dashed by delays and limits on public access to many types of data.

### Cape Cod study.

The Cape Cod Breast Cancer and Environment Study also began from a legislative mandate (General Court of the Commonwealth of Massachusetts 1994), although at the state rather than federal level. In response to Massachusetts Department of Public Health (MDPH) data showing elevated incidence on Cape Cod (Brody et al. 1996), MBCC founded Silent Spring Institute in 1994 to bid for and win a $1 million annual state appropriation for breast cancer and environment research. The institute’s researchers in epidemiology, toxicology, and environmental science now collaborate with co-investigators from Boston, Brown, Harvard, and Tufts universities and elsewhere.

The founders’ vision transcended “science as usual” and gave activists governance roles on the scientific team. As a nonprofit organization, the institute has a public-interest board of directors (including three directors chosen as MBCC representatives) with the authority to hire and fire the study’s principal investigator. The board’s authority is tempered by grant requirements for funder approval before key personnel can be replaced, however, and the Silent Spring Institute board developed additional mechanisms to ensure that it exercises its authority responsibly. The board convened a science advisory committee of outside experts, frequently sent a representative to co-investigator meetings, gave added weight to input from board members trained in biology and medicine, and supported publication of research results in peer-reviewed scientific journals even when MDPH disallowed use of state funds for this purpose. This activist-governed research model is particularly notable at a time when government increasingly relies on industry science in regulatory decisions and academic science is growing more dependent on industry funding (Krimsky 2003). Further, breast cancer activists often cite their hope of putting themselves out of business by finding scientific answers to “stop the epidemic,” so their governance role may help check any possible bias stemming from researchers’ interests in perpetuating their own work.

### NIEHS Centers.

The NIEHS Centers program began as an initiative of the National Breast Cancer Coalition, which, along with NIEHS, convened a series of invitational brainstorming sessions for researchers and activists. These sessions, coupled with public meetings, shaped the request for applications (RFA). The RFA specified a multidisciplinary approach, including laboratory and epidemiologic components, and required ongoing public involvement (NIEHS/National Cancer Institute 2002).

Among these three examples of activist-initiated research, the Cape Cod and NIEHS models have extended the democratization of science in ways that can offer models for the development of new norms for environmental health research: public empowerment that goes beyond mere involvement on advisory boards, a shift away from purely investigator-defined research to joint activist–scientist definition of research problems, and integration across disciplines and across institutions. The Cape Cod study is perhaps unique even in the history of community-based participatory research in that activists govern the research team.

## Research Questions and Study Design

If democratization in science makes a difference, we would expect to see activist-initiated studies that differ in design from the typical investigator-initiated studies funded by the National Cancer Institute, Department of Defense, and major foundations. Consistent with breast cancer activists’ support for the pre-cautionary principle, we expect study designs that will inform preventive public health policies in the face of uncertainty [the first principle of the Wingspread Statement on the Precautionary Principle (Raffensperger and Tickner 1999)]. The research that serves this goal includes assessments of such factors as “upstream” health outcomes (e.g., precursors of disease), multiple sources of uncertainty in measurements and models, effects on sensitive individuals, the nature and effects of high exposures, exposure pathways, cumulative and interactive effects of multiple exposures, population as well as individual effects, and the environmental justice implications of the distribution of health risks across exposure levels and across populations (Kriebel et al. 2001; Stirling and Gee 2002; Tickner 2002, 2003). We add to this list of relevant research activities the development and application of animal and cell models that can inform understanding of natural systems and the plausibility of effects in humans (Brody and Rudel 2003). If EDCs make breast cancer cells grow in the laboratory, for example, they may also affect breast cancer in women. Animal and cell studies are particularly valuable when human studies are technically or ethically difficult to undertake.

### Long Island study.

The Breast Cancer and the Environment on Long Island case–control study, the centerpiece of the LIBCSP, applied a typical hypothesis-testing framework to investigate whether an association exists between breast cancer risk and organochlorine compounds [dichlorodiphenyltrichloroethane (DDT)/dichlorodiphenyldichloroethylene (DDE), chlordane, dieldrin, and polychlorinated biphenyl], which are EDCs, and poly-cyclic aromatic hydrocarbons (PAHs), which are mammary carcinogens (Gammon et al. 2002a, 2002b). From the perspective of a pre-cautionary science model, the choice of exposures for study is mixed. The organochlorine compounds are banned in the United States, so findings are not directly actionable, but if the study had shown an effect, it would have strengthened the existing evidence from studies of pharmaceutical estrogens that exogenous hormones contribute to breast cancer, adding support for precautionary action regarding other EDCs. PAHs—the source of ubiquitous and avoidable exposure from grilled and smoked foods, tobacco smoke, and air pollution from vehicle exhaust and other fossil fuel burning—have clear action implications.

Aside from the choice of target compounds, case–control studies can serve public health decision making by generating an estimate of relative risk and its confidence interval. However, we consider this a high-risk strategy in both the Long Island and Cape Cod studies from a precautionary perspective, because of the considerable expense coupled with the likelihood of generating inconclusive negative findings, which are common in case–control studies of hard-to-assess exposures to pollutants in the general population. Several factors favored the potential in the LIBCSP to produce persuasive evidence that organochlorines increase breast cancer risk: the biologically plausible hypothesis that EDCs affect breast cancer, several earlier studies showing an association between breast cancer and serum organochlorines, a large sample size (providing good statistical power to detect an effect), rapid case ascertainment (so serum measures could not be affected by breast cancer treatment), extensive interviews about established and hypothesized breast cancer risk factors (to control for confounding and investigate effect modification), and individual-level biologic markers of exposure. On the other hand, results that failed to show an association could contribute little, because study design limitations mean we cannot conclude from null results that no association exists. For example, no one in the study can reasonably be considered unexposed, raising questions about whether there is adequate exposure variability to detect effects. In addition, the one-time exposure measures do not accord well with the evidence that timing in the life cycle is important in breast cancer etiology. Specifically, serum measures taken near the time of diagnosis may not represent early life exposures or even total lifetime exposure, because recent levels are influenced by variables related to mobilization and excretion, such as weight gain/loss and lactation, and by intake of breakdown products in food that have different toxicologic properties from the parent compound (e.g., DDE, which is ingested in meat and dairy, is less estrogenic than the parent compound DDT) (Brody and Rudel 2003; Snedeker 2001). Results did not show an association between recent serum measures and breast cancer (Gammon et al. 2002b).

The Long Island study reported 50% higher breast cancer risk among women with the highest levels of DNA damage from selected PAHs, statistically significant at the traditional *p* < 0.05 level, but with no linear dose response (Gammon et al. 2002a). It now falls to the public and policy makers to evaluate whether this result supports precautionary steps to reduce exposure, particularly in light of other evidence of health damage from PAHs and available alternatives to reduce exposure. This decision is hindered, however, because the biologic exposure measure does not reveal the exposure source where policies might be designed to intervene. The DNA adduct measure was poorly correlated with self-reported dietary and tobacco sources, leaving us to speculate that air pollutants may be an important source. It is also useful to consider the policy implications if air pollutants are an important source. The study’s effect size—50% higher breast cancer risk with high PAH DNA damage—is sometimes considered small in epidemiology but is nevertheless larger than the estimated 30% reduction in mortality associated with regular mammogram screening (Nystrom et al. 2002; Olsen and Gotzsche 2001). Epidemiologists have good reason to be cautious about a relatively modest risk increase observed in a single study with a poorly understood exposure measure. Given the potentially enormous public health implications, however, we believe a substantial investment in follow-up is appropriate.

Follow-up research currently under way is investigating possible interactions between exposure and genetic susceptibility. This approach is consistent with the precautionary principle call to study vulnerable populations, and it may yield additional information of value for prevention.

### Cape Cod study.

In the Cape Cod study, the activists’ request for state funds for an unusual 3-year scoping and planning process helped define the research questions. During this phase, the study team formed a public advisory committee and a scientific advisory committee, established a field office on Cape Cod, and conducted focus groups that included physicians, nurses, women with breast cancer, and long-time residents. We reviewed scientific literature, analyzed existing Cape Cod environmental and epidemiologic data, conducted pilot environmental studies, and developed new methodologies suited to the nascent research questions.

This process provided an opportunity for the convergence of public and scientific priorities. Usually, study questions and protocols are defined by researchers (in investigator-initiated programs) or by funding agencies (in RFAs). Thus, the development of the research ideas, goals, and methods precedes formal funding of the study, making it more difficult for scientists and the community members to debate together the research agenda at this crucial design stage.

The Cape Cod study team reviewed nine issue areas—ranging from local food distribution systems to military facilities—as candidates for study and set priorities based on three criteria: scientific literature showing a plausible link to breast cancer; evidence of exposure on Cape Cod, particularly distinctive exposure; and community concern. Scientific evidence included laboratory studies of animal models and cellular mechanisms and epidemiologic studies. These criteria and types of evidence provide widely applicable guidelines for selecting research questions under the precautionary principle, because they emphasize assessing plausibility in situations in which proof is unlikely to be achievable. Including community concern as a decision-making criterion helps avoid studies that, although elegantly designed, do not answer relevant questions, a pitfall sometimes referred to as a type III error (Tickner 2003).

The scoping process also incorporated surveillance and ecologic epidemiology to refine the definition of the problem and generate hypotheses. This process illustrates how a precautionary approach can generate evidence that appropriately reduces public concern in some areas and focuses attention on more promising hypotheses. Using GIS technology, we integrated breast cancer and environmental data and searched for geographic and temporal patterns. We geocoded home addresses from the Massachusetts Cancer Registry of about 2,600 Cape Cod women diagnosed between 1982 and 1994 and used U.S. Census data and population growth models to estimate age-adjusted standardized incidence ratios annually by census block group (Silent Spring Institute 1997).

Results showed consistently higher incidence rates on Cape Cod than in the rest of the state; rates of “early” stage 1 diagnosis and mammography could not account for the higher incidence rates (Silent Spring Institute 1997, 2004). Mapping revealed that exposure of residences to electromagnetic fields (EMFs) from power lines was uncommon and regional high incidence was not localized around the military reservation or nuclear power plant. These population-level analyses confirmed suspicions that elevated breast cancer risk on Cape Cod was significant and long-standing; refocused public attention away from the military reservation, nuclear plant, and power line EMFs as the cause; and developed the GIS that would later be used for individual-level exposure assessments.

Phase 1 also included an innovative field study of EDCs in Cape Cod wastewater, groundwater, and drinking water to assess the plausibility of exposure from drinking water wells affected by septic systems. This aspect of the study had several characteristics designed to meet community precautionary goals. It was small in scope, with 12 groundwater and wastewater samples and 28 drinking water samples designed to assess plausibility rather than to establish representative results. It cast a broad net by testing for 29 target compounds; was integrative in that it used an *in vitro* bioassay of estrogen-sensitive cells—the E-Screen bioassay—to assess total estrogenicity (Soto et al. 1995); and used low detection limits, often below regulatory thresholds. The study contributed to a new field of inquiry by reporting the first measurements of estrogenic activity in groundwater, supplementing previous research on surface water (Silent Spring Institute 1997). And the study had local as well as national significance because land use and wastewater management policies to protect drinking water are under active discussion on Cape Cod. Results showed high levels of estrogenic alkylphenols in wastewater and groundwater and low levels in a small number of private wells, documenting an exposure pathway through drinking water (Rudel et al. 1998).

During phase 1, the study team updated community members and local officials through quarterly meetings of the public advisory committee, legislative briefings, and “poster sessions” where scientists and community members could interact informally to respond to community concerns. At the close of phase 1, the scientific team prepared technical and lay documentation and atlases of health and environmental data (Silent Spring Institute 2004). The drinking-water quality data page in the atlas has become the second most visited page in the Silent Spring Institute website, which hosts 400,000 visits per year. Based on the phase 1 assessment, the study team recommended further investigation of EDCs, particularly from wide-area pesticide use and wastewater-contaminated drinking water.

The second phase began in 1997 with a new competitive bidding process in which MDPH specified a cohort or case–control study (MDPH 1997), although the 3-year time frame argued against a cohort study. Silent Spring Institute won funding for a case–control epidemiologic study, which ultimately included 2,100 Cape Cod women and an environmental sampling study of 89 EDCs in air, dust, and women’s urine from 120 homes. Negotiation of the final study protocol revealed contrasting perspectives between the activist-scientist team and MDPH. For example, MDPH required that the proposed research questions be recast as statements of null hypotheses, a more yes-or-no approach than the study team thought best fit the state of the science. The state also declined to fund research in a comparison geographic area off Cape Cod—a decision, perhaps motivated mostly by cost concerns, that fundamentally precluded answering the public’s original question: Why is breast cancer incidence higher on Cape Cod? Other proposed elements that were not funded included soil sampling to validate the GIS-based pesticide exposure estimates (Brody et al. 2002) and additional testing of groundwater and drinking water to follow up on phase 1 findings of high concentrations of EDCs in groundwater, a research area with potentially far-reaching and expensive public health policy implications.

Nevertheless, the study retained many elements of a scientific approach focused on pre-cautionary strength-of-evidence goals. The study’s scientific publications have addressed seven core research questions, more than half of which focus on exposure assessment: *a*) What is the history of exposure to wastewater contaminants (particularly EDCs) in public and private drinking water (Swartz et al. 2003)? *b*) What is the history of exposure to pesticides from wide-area application (Brody et al. 2002)? *c*) What EDCs are women exposed to at home (Rudel et al. 2003)? *d*) How do EDCs from septic systems travel in groundwater, which supplies drinking water (Rudel et al. 1998)? *e*) After controlling for established risk factors, is living longer on Cape Cod associated with breast cancer risk (McKelvey et al. 2003)? *f* ) Is exposure to pesticides from wide-area application associated with breast cancer risk (Brody et al. 2004)? *g*) Is exposure to drinking water contaminants associated with breast cancer risk (Brody JG, Aschengrau A, McKelvey W, et al., unpublished observations)?

The exposure questions are key because they are critical in assessing plausibility (without exposure to a causal agent, there is no health effect), they are prerequisite to health studies, and they identify preventable exposures that could be reduced by precautionary policies, even in the absence of strong evidence of harm.

Ideally, a breast cancer study would estimate exposures years before diagnosis and at particular times in the life cycle. Retrospective self-reporting can offer this standard for exposures that women themselves can identify and are likely to report without bias, such as the year and their age at the births of their children, which reveals that pregnancy within 5 years of diagnosis and older age at the birth of a first child both increase breast cancer risk (Bernstein 2002). To approach this goal for environmental exposures that women cannot report, Silent Spring Institute developed GIS methods to map pesticide drift and drinking water contamination from historical records (Brody et al. 2002; Swartz et al. 2003) and incorporated these assessments with interview data (Brody et al. 2004). We also estimated the consequences of uncertainty in the exposure assessment by using sensitivity analyses. Missing environmental data and a lack of precision in address histories form the primary limitations in GIS exposure assessments, so future studies could be strengthened by the systematic geographic tracking of environmental data and the ascertainment of address histories at the time of reportable diagnoses, such as cancer (Hurley et al. 2003; Wakefield 2000).

Although the GIS exposure assessment is valuable for developing new methods for public health studies, its application in the Cape Cod study shares with the Long Island study the risk of generating findings that are difficult to interpret because of uncertainties in the exposure assessment. Indeed, the results have been ambiguous. We found no consistent association between pesticides and breast cancer and weak evidence of associations with certain types of pesticide use (Brody et al. 2004). After controlling for established breast cancer risk factors, however, we did find that living longer on Cape Cod is significantly associated with higher breast cancer risk (McKelvey et al. 2003). This “black box epidemiology” (Greenland et al. 2004) result provides convincing evidence that an additional regional risk factor remains to be discovered but offers no further guidance on where to look.

Parallel to the drinking water sampling in phase 1, phase 2 included monitoring of EDC exposures in homes with these goals: identify common exposures, including mixtures, for toxicologic and epidemiologic study and regulation; identify the products or practices that lead to common exposures; identify factors that contribute to high-end exposures; test methods to reduce contaminant levels by changing product use and other practices; and develop methods of exposure assessment for future health studies.

The household exposure study has not been linked to health outcomes in the epidemiologic study because of low statistical power for that purpose, and information on the health significance of these exposures is not available. This strategy of broadly studying exposure without an identified health outcome is atypical in public health studies—perhaps because health officials are uncomfortable dealing with the uncertain action implications of reporting on exposure without an established tie to health—but it has received strong scientific and public interest (e.g., Betts 2003; Cone 2003). This approach produced the first reported indoor concentrations for 30 pollutants and data directly relevant to public health debates, such as the use of polybrominated diphenyl ethers as flame retardants.

State funding for environmental sampling in the Cape Cod study resulted from advocacy by MBCC, and breast cancer activist organizations also have provided financial support for the work. Recently, the household exposure study has become a point of connection between breast cancer advocacy and other health-affected groups. For example, the study team is currently collaborating with Brown University researchers and Communities for a Better Environment (Oakland, CA), a community-based environmental justice organization, to apply the methods in a low-income, ethnic-minority fenceline community, where the immediate focus will be on whether exposure data can be useful in evaluating emissions limits, flare rules, and emergency procedures.

### NIEHS Centers.

Still in a relatively early stage of development, the NIEHS Breast Cancer Centers and the Environment Research Centers were initiated with several important elements consistent with the precautionary principle. First, the RFA specified girls’ development through puberty as the health outcome, which represents a breakthrough in moving “upstream” in breast cancer research. Early age at puberty is a well-established risk factor for breast cancer, and age at puberty is falling, particularly among African-American girls, a group at greater risk than whites for breast cancer mortality, though not incidence (Bernstein et al. 2003; Krieger 2002). In addition, researchers hypothesize that rapid breast cell proliferation during adolescence may make this a critical exposure period. Thus, research questions about adolescence resonate with the precautionary principle because they address vulnerable populations, allow investigation of subtle and complex phenomena, and contribute to the understanding of the natural development process.

By including a laboratory research component as well, the centers elucidate biologic mechanisms, an important element in assessing plausibility, and develop tools for screening and testing chemicals for possible regulation. The laboratory component also facilitates research on a longer list of chemicals than the epidemiologic study. The RFA did not, however, specifically call for an investment in exposure assessment, although the lack of such methods is a significant barrier to studying EDCs (Rudel et al. 1998, 2001). The epidemiologic study will evaluate the EDCs bisphenol A, dioxin, and di(2-ethylhexyl) phthalate, as well as individual factors such as diet and body size. The study will bank biologic specimens, an increasingly common practice, so that researchers can “try again” as science advances, a strategy that may improve the payback on investments in large epidemiologic studies.

The centers’ steering committee, composed of scientist and advocate representatives, integrates epidemiologic and laboratory work, scientist and activist perspectives, and the interests of the different centers. This management approach represents innovation in both science and public involvement. The centers program recently held its first scientist–advocate conference (Russo 2004), at which both scientists and breast cancer activists were session chairs and presenters.

## Conclusion

As the continuing increase in breast cancer incidence sparked activist demands for prevention-oriented research, laboratory evidence that many common pollutants are mammary carcinogens and/or EDCs provided new hypotheses about environmental factors. But the challenges in assessing relevant exposures to pollutants in a breast cancer study meant a mismatch between activist goals and the scientific methods typically used in investigator-initiated epidemiologic studies. By examining recent research—the Long Island and Cape Cod breast cancer and environment studies and the new NIEHS Centers—we can draw lessons for many public health problems for which scientific uncertainty is likely to persist.

Each of these studies contributes novel public involvement methods and increases transparency in public health science, providing new models for community-based participatory research. Activists used legislation and appropriations processes to direct scientific inquiry and, in Massachusetts, founded the Silent Spring Institute as a scientific team with activist participation in governance. The 3-year scoping process in the Cape Cod study provided an opportunity to review scientific plausibility of multiple hypotheses, allowing activist and scientist perspectives to converge.

Far from hindering science, the involvement of breast cancer activists has helped drive scientific innovation, particularly in the development and application of exposure assessment methods. Environmental and biologic sampling methods can identify common mixtures for further study and inform precautionary exposure reduction. GIS methods can assess historical exposures that women cannot report. The suggestive positive result for PAHs in the Long Island study provides the impetus for policies to reduce ubiquitous PAH exposure. At the same time, however, unresolved weaknesses in exposure assessment methodologies have hindered the success of epidemiologic components of the research programs, because they mean that negative results are insufficient to conclude that no relationship exists.

Breast cancer activists were among the first and most powerful health-affected groups to make environmental research and prevention a priority. The resulting studies provide paradigmatic models for public health science for diseases whose links to environmental factors are difficult to prove. They argue for greater emphasis on exposure studies before undertaking health studies and on laboratory research on questions that do not lend themselves to human research. Yet they leave unanswered questions about when to invest in traditional epidemiologic studies, when negative results are sufficient, and how to pursue ambiguous positive results in further research and policy.
